# Integrated control strategies for dengue, Zika, and Chikungunya virus infections

**DOI:** 10.3389/fimmu.2023.1281667

**Published:** 2023-12-18

**Authors:** Nelson Côrtes, Aline Lira, Wasim Prates-Syed, Jaqueline Dinis Silva, Larissa Vuitika, William Cabral-Miranda, Ricardo Durães-Carvalho, Andrea Balan, Otavio Cabral-Marques, Gustavo Cabral-Miranda

**Affiliations:** ^1^ Department of Immunology, Institute of Biomedical Sciences, University of São Paulo, São Paulo, Brazil; ^2^ The Interunits Graduate Program in Biotechnology of the University of São Paulo, the Butantan Institute and the Technological Research Institute of the State of São Paulo, São Paulo, Brazil; ^3^ The Graduate Program in Pathophysiology and Toxicology, Department of Clinical and Toxicological Analyses, School of Pharmaceutical Sciences, University of São Paulo, São Paulo, Brazil; ^4^ Institute of Research and Education in Child Health (PENSI), São Paulo, Brazil; ^5^ São Paulo School of Medicine, Department of Microbiology, Immunology and Parasitology, Federal University of São Paulo, São Paulo, Brazil; ^6^ Applied Structural Biology Laboratory, Institute of Biomedical Sciences, University of São Paulo, São Paulo, Brazil; ^7^ Department of Medicine, Division of Molecular Medicine, University of São Paulo School of Medicine, São Paulo, Brazil

**Keywords:** arboviruses, tropical regions, climate change, dengue virus, Zika virus, Chikungunya virus, strategies control, public health

## Abstract

Arboviruses are a major threat to public health in tropical regions, encompassing over 534 distinct species, with 134 capable of causing diseases in humans. These viruses are transmitted through arthropod vectors that cause symptoms such as fever, headache, joint pains, and rash, in addition to more serious cases that can lead to death. Among the arboviruses, dengue virus stands out as the most prevalent, annually affecting approximately 16.2 million individuals solely in the Americas. Furthermore, the re-emergence of the Zika virus and the recurrent outbreaks of chikungunya in Africa, Asia, Europe, and the Americas, with one million cases reported annually, underscore the urgency of addressing this public health challenge. In this manuscript we discuss the epidemiology, viral structure, pathogenicity and integrated control strategies to combat arboviruses, and the most used tools, such as vaccines, monoclonal antibodies, treatment, etc., in addition to presenting future perspectives for the control of arboviruses. Currently, specific medications for treating arbovirus infections are lacking, and symptom management remains the primary approach. However, promising advancements have been made in certain treatments, such as Chloroquine, Niclosamide, and Isatin derivatives, which have demonstrated notable antiviral properties against these arboviruses *in vitro* and *in vivo* experiments. Additionally, various strategies within vector control approaches have shown significant promise in reducing arbovirus transmission rates. These encompass public education initiatives, targeted insecticide applications, and innovative approaches like manipulating mosquito bacterial symbionts, such as *Wolbachia*. In conclusion, combatting the global threat of arbovirus diseases needs a comprehensive approach integrating antiviral research, vaccination, and vector control. The continued efforts of research communities, alongside collaborative partnerships with public health authorities, are imperative to effectively address and mitigate the impact of these arboviral infections on public health worldwide.

## 1 Introduction

The arbovirus (arthropod-borne viral) diseases have become serious public health problem with a wide geographic distribution, mainly in tropical regions (1), due to the presence of competent vectors (2). These infectious agents predominantly contain ribonucleic acid (RNA) as their genetic material, with an exception that is the *Asfarviridae* family, which have deoxyribonucleic acid (DNA) as their genetic material (3).

**Figure 1:**
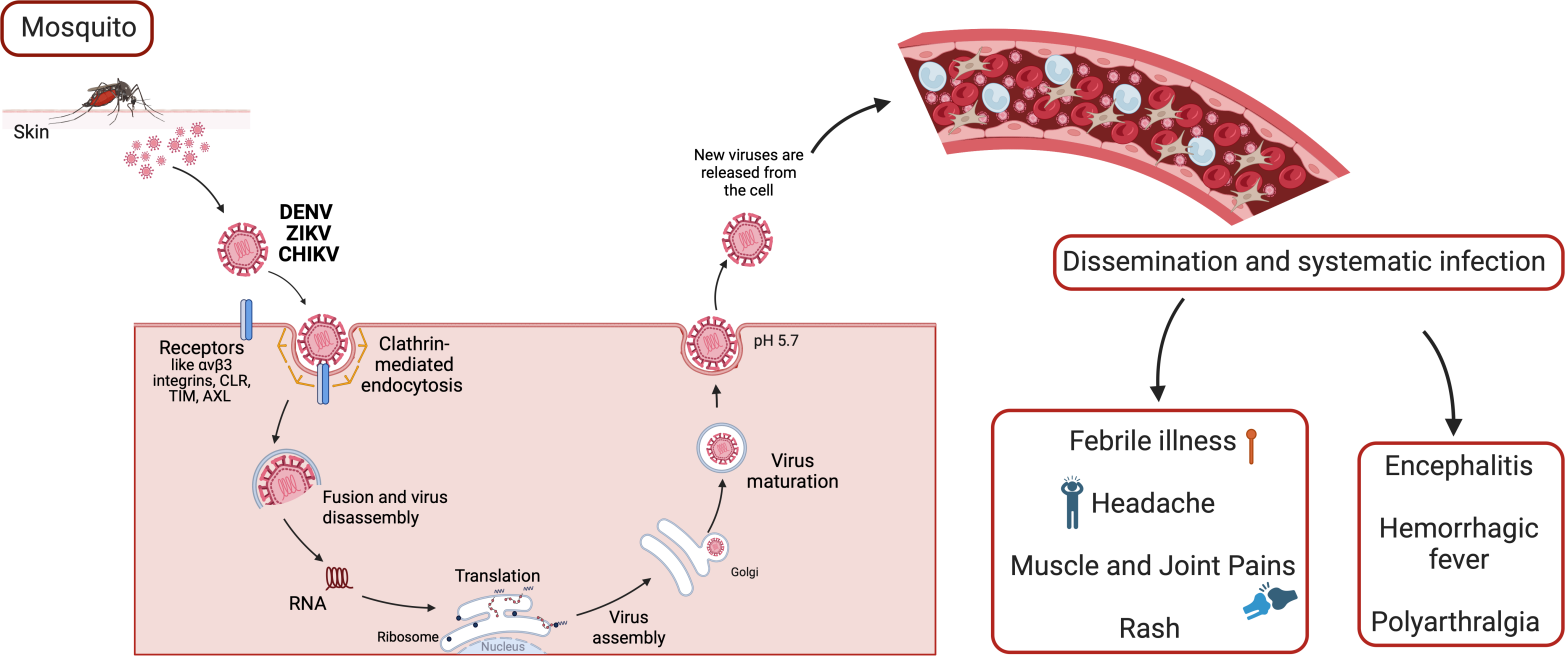
The arbovirus replication cycle and systematic infection. Arboviruses interact with multiple types of host attachment factors, including molecules that bind to the viral membrane or virion-associated N-linked carbohydrates. Virions are internalized by clathrin-dependent mechanisms that usurp host factors involved in the uptake of large macromolecules. Subsequently, viral RNA replication takes place, and infected cells migrate through skin into blood stream and lymphoid organs, such as lymph nodes, spleen, liver and brain. Host immune response, and each affected organ can trigger different sinptoms and pathologies.

At the moment, there are more than 534 arboviruses identified, of which approximately 134 cause disease in humans (4). The families of arboviruses include *Flaviviridae*, *Togaviridae*, *Asfarviridae, Orthomyxoviridae, Reoviridae, Rhabdoviridae and Bunyaviridae* (5, 6). Most arboviruses are asymptomatic, but those of medical interest often cause symptoms (**Figure 1**) such as febrile illness, headache, muscle and joint pains. Rash may be present, as well as petechial rashes. In some cases, arboviruses can cause encephalitis, hemorrhagic fever, or polyarthralgia (6).

Dengue virus (DENV) is the most prevalent arbovirus worldwide, found in more than 100 tropical and subtropical countries (7). According to the World Health Organization (WHO), it affects approximately 16.2 million people annually only in the Americas, with more than 500 million people at risk of contracting the disease in this region. As reported by the Pan American Health Organization (PAHO), in 2022, more than 2.8 million cases were registered in the Americas, with 4,607 of them being serious cases that caused 1,292 deaths. It is estimated that half of the world’s population is at risk of contracting the disease (8).

There are four genetically distinct serotypes of DENV that cause the dengue disease (9). However, in 2013, a fifth serotype was first detected in the blood of a patient in the state of Sarawak, Malaysia (10). In endemic countries, there can be co-circulation of more than one DENV serotype, as well as other arborviruses, all mainly transmitted by mosquitoes of the *Aedes* genus (11).

DENV-1 was first isolated in 1943 in Japan and later in 1945 in Hawaii. Since 1977, some countries in America have reported cases of DENV-1 (12–14). Over the years, new epidemics have emerged in several countries, leading to the reporting of other DENV serotypes. In 1944, DENV-2 was reported in Papua New Guinea and Indonesia, and in 1953 in Americas. In the 1970s, it started to spread to countries in Latin America, such as Puerto Rico and Brazil in 1984. DENV-3 and DENV-4 were first reported in 1953 in the Philippines and Thailand. The reports of DENV-3 in Brazil started around 2000s in Rio de Janeiro, and DENV-4 in 1981 (11, 14, 15).

The Zika virus (ZIKV) is another re-emerging flavivirus and human pathogen, that was first isolated in 1947 in the Zika forest in Uganda (16). Although the ZIKV has many similarity with other flavivirus, such as DENV, the ZIKV has an highly variable region present in its envelope, located close to an important glycosylation site that contains the amino acid asparagine at position 154 (Asn154) which is associated with its virulence (17). Before 2007, it was identified as a virus that cause a mild feverish disease in a small number of humans in Africa and parts of Asia. However, ZIKV has since spread to several countries, and in 2014, it was introduced in Brazil and other regions in the Americas (6). Genomic and phylogenetic analysis of virus isolates indicated the presence of two distinct lineages, African and Asian, both originating in East Africa (18). In addition to viral transmission through the bite of the *Aedes* mosquitoes, there are other forms, such as transmission through infected blood transfusion, sexual, and maternal-fetal transmission (19) which makes it even more difficult to control.

The chikungunya virus (CHIKV) was initially identified in patient serum during an epidemic in 1952 in Tanzania, and in 1953, the virus was isolated from *Aedes* and *Culex* spp. mosquitoes (20, 21). Phylogenetic studies show that the circulating strains of CHIKV have a common ancestor that originated 500 years ago (22). Currently, there are four identified CHIKV lineages, namely the Asian (AL), West African (WA), East/Central/South African (ECSA), and Indian Ocean (IOL) lineages. Among these, two main strains, responsible for several epidemics in Brazil, are the ECSA and AL (23, 24).

The ECSA genomic sequences isolated from patients of the outbreak in ‘La Reunion Island in the Indian Ocean’ showed a mutation in 90% of the viral sequences of the structural protein E1, where there was an exchange of the amino acid alanine for valine in region 226 (A226V) (25, 26). This mutation allowed the adaptation of a new vector, *A. albopictus*, which is present in temperate regions, favoring outbreaks in Italy and France (25, 26). A seroprevalence analysis between the years 2000-2019 found that the ECSA strain is the most frequent (27).

Since the first record of CHIKV in 1950s, several epidemics have emerged in Africa, Asia, Europe, and the Americas. Nowdays, CHIKV has been found in more than 100 countries (28). According to the WHO, more than one million cases are reported annually only in the Americas, with most cases occuring in Brazil (29). In 2016, in the Caribbean and the Americas, 185,000 cases of CHIKV were reported, with more than 90% of cases in the Americas being in Brazil (29).

**Figure 2:**
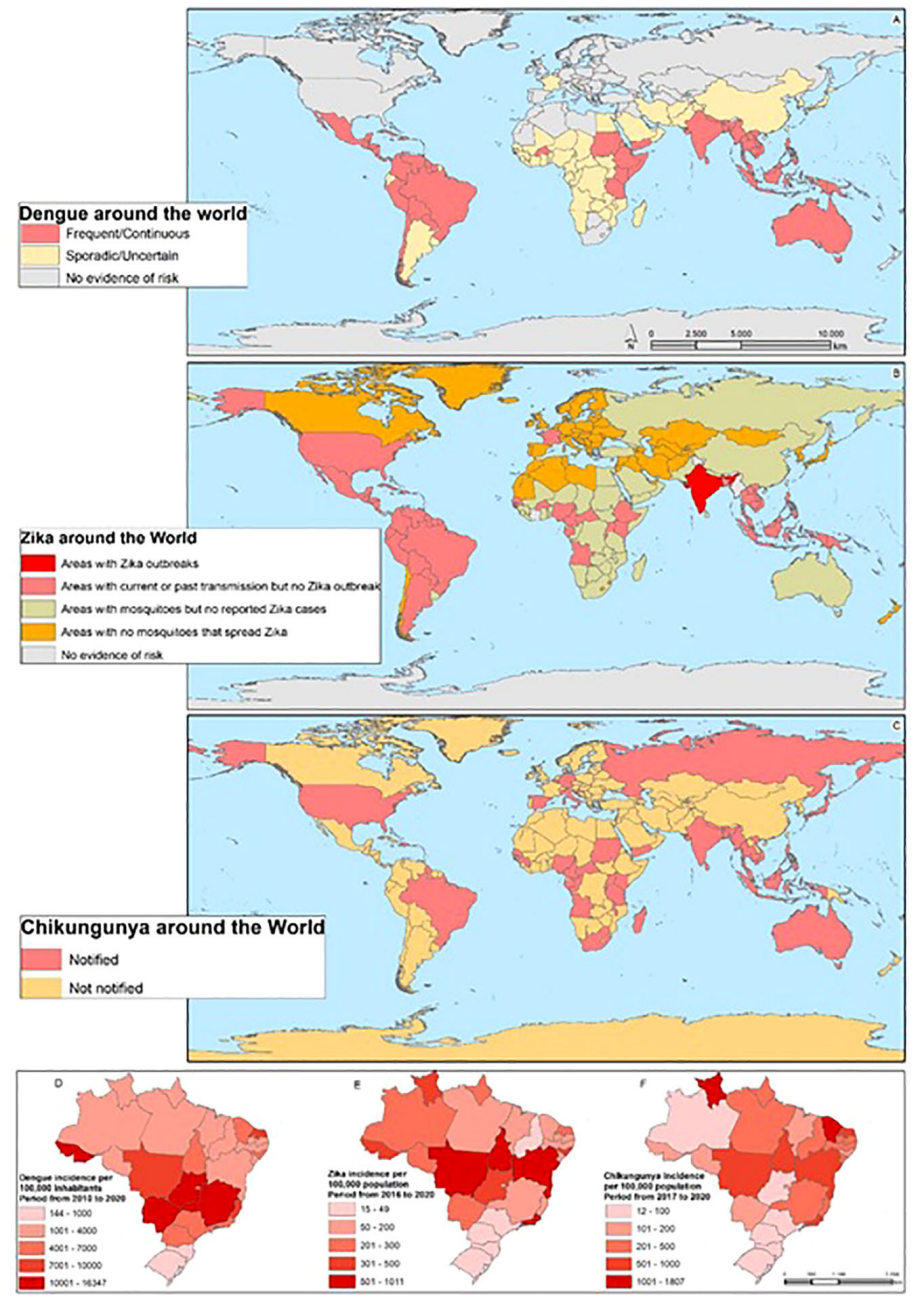
The Arbovirus DENV, ZIKV, and CHIKV distribution around the world: The map shows the areas with the distribution of the risk of DENV throughout the world **(A)**, the areas with the risk of ZIKV **(B)**, and the areas of the risk of CHIKV **(C)**. On **(D)**, the incidence of DENV is shown in the Brazilian states, **(E)** ZIKV, and **(F)** CHIKV.

The geographic distribution (**Figure 2**) of each arbovirus is related to ecological parameters that define the transmission cycle. Some factors include patterns of vegetation, temperature, and typical precipitation, which influence the distribution of the arthropod vector and the vertebrate host necessary for the maintenance of the virus. Social, demographic and climate change, deforestation, population migration, and urbanization in recent years have also had a strong impact on arbovirus infections (30).

All these factors increase the possibility of human contact with vectors, contributing to the increasing transmission of viruses and the appearance of epidemics around the world. In recent decades, for example, the reemergence of arboviral infections has been observed in numerous countries (**Figure 2**). The reemergence of CHIKV in Asia, ZIKV outbreaks in the Americas, as well as West Nile virus (WNV) and Rift Valley fever virus (RVFV) outbreaks in Europe, are increasingly frequent (15, 31–33).

Therefore, control and prevention programs for arboviruses are essential, which must be based on the development of vaccines, treatment, vector control, and genomic surveillance programs based on tracking viruses using genomic sequence data as a cross-cutting activity (34). In this manuscript, we discuss the principal arboviruses, such as DENV, ZIKV and CHIKV, responsible for outbreaks in many countries. Furthermore, we address the perspectives of controlling these diseases.

## 2 Viral structure and morphogenesis

**Figure 3:**
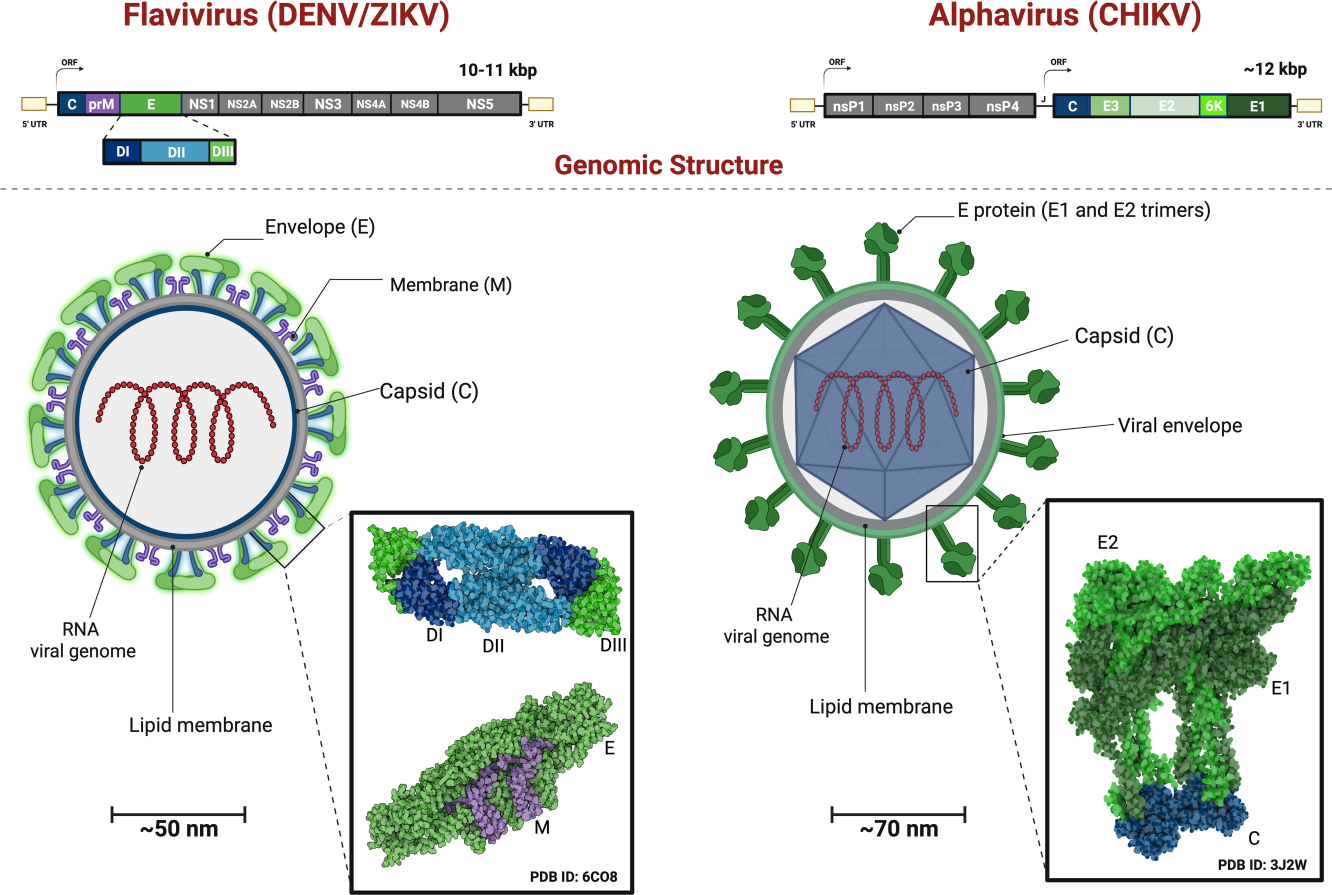
Genomic Organization and Viral Structure of Flavivirus and Alphavirus. Flavivirus comprise a single Open Reading Frame with the genes for the Structural Proteins followed by the Non-Structural Proteins transcribed and translated and the resulting polyprotein undergoes proteolytic processing. Alphavirusus are comprised of the genes for the Non-Structural Proteins followed by the Structural Proteins, transcribed from two distinct ORFs, and each resulting polyprotein undergoes further proteolytical processing. Flavivirus structure have a mature virions with capsid (C) and membrane (M) covered by 90 dimers of (E) proteins. The E proteins exhibit three domain: E-DI (dark blue), E-DII (light blue) and E-DIII (green) anchored to the viral membrane (purple). The Alphavirus virion have the envelope glycoproteins in a shape of 240 dr trimer of E1(dark green), E2 (light green) ancored in lipid membrane (M) and the capsid (C) protein (blue). Showing heterodimers interacting with capsid protein.

Flaviviruses like DENV and ZIKV have a spherical and small composition of viral particles (~50nm in diameter). They contain a 10-11 kb genome and an open reading frame (ORFs) that encodes three structural proteins: capsid (C), premembrane/membrane (prM/M), and envelope (E), as well as seven non-structural proteins (NS1, NS2A, NS2B, NS3, NS4A, NS4B, and NS5), as seen in the **Figure 3**. The structural and non-structural proteins are important for correct virion assembly, cell receptor binding, and viral replication (**Figure 1**) (12, 35). The E protein is an essential structure responsible for viral attachment and membrane fusion. It is composed by three domains (E-DI, E-DII, and E-DIII) linked to the viral membrane by a helical region and two antiparallel transmembrane segments. The E-DI is responsible for the structural organization of the envelope, while E-DII allows the fusion of the virus with the host cell membrane (36, 37), and the E-DIII is highlighted as the most neutralizing antibody site and allows the virus to bind to the cell receptor (38, 39). Therefore, the E-DIII has been used as the best candidate for vaccine development, as well as for treatment (40).

The viral capsid (C) is a small helical protein with surfaces that bind to viral nucleic acids or host lipids and directs the incorporation of the viral genome into the host cells (17). The M protein originates from the cleavage of the precursor protein prM (41) and it is essential for the maturation of virions. For example, in the immature virions, prM prevents structural changes in the E glycoprotein, which are essential for the correct folding, maturation, and assembly of E protein during replication (**Figure 1**) (42).

As illustrated in **Figure 3**, the alphaviruses like CHIKV form spherical particles (~70nm in diameter) with icosahedral symmetry in their capsids. The virions consist of a host-derived lipid bilayer that is enriched in cholesterol. The lipid envelope contains 240 copies of E1 and E2 heterodimers glycoproteins arranged in a trimeric format (80 copies) (43, 44). The genome consists of two ORFs that encode four nonstructural proteins (nsP1-nsP4) and six structural proteins (Capsid, E3, E2, 6K, E1).

Some receptors involved are the Mxra8, αVβ3 integrins, C-type lectin receptors (CLR), TIM, TAM, AXL and phosphatidylserine receptor families (T cell immunoglobulin and mucin domain) (45). Most of the *Arbovirus* enter the cells by clathrin-mediated endocytosis (12, 46, 47). In the case of flaviviruses, the viral polyprotein (NS2-NS3) undergoes cleavage in the endoplasmic reticulum (ER) by host proteases to produce functional proteins. The viral genome is synthesized within a viral capsid (C) and surrounded by a lipid bilayer containing two transmembrane proteins, envelope (E) and membrane (M). Subsequently, the particles are released from the ER (**Figure 1**) (35).

In the case of alphaviruses, the E1 glycoprotein fuses the viral envelope in the endosome, while E2 interacts with the cell receptor, triggering endocytosis. The E2 glycoprotein conjugates with E3 glycoprotein (pE2) inside the cell. pE2 undergoes maturation in the Golgi complex and releases E2 and E3. Cleavage of E3 is crucial for particle fusion. E3 aids in spicule folding and prevents premature glycoprotein activation. 6K is involved in virion formation and growth (43, 48, 49). Nonstructural proteins play essential roles in genome replication and transcription, as well as in protecting the replication complex and genomic RNA from protease degradation (38). These proteins are translated into two forms: P123, which is more abundant, and P1234, generated by reading the opal stop codon at the junction of nsP3 and nsP4. Following cleavage, these proteins give rise to four distinct proteins: nsP1, nsP2, nsP3, and nsP4. The nonstructural proteins actively participate in the replication of the viral genome (50–55).

After attachment to the host cells, mainly innate immune cells, such as dendritic cells, monocytes, and macrophages, by receptor-mediated endocytosis, the viral RNA replicates. Once infected, dendritic cells migrate to lymphoid organs, allowing replication and dissemination to other organs (**Figure 1**) (56).

## 3 Pathogenicity of arboviruses and host immune responses

The arbovirus infects resident skin cells, such as fibroblasts, macrophages, and dendritic cells, through the bite of an infected female *Aedes* spp. mosquito (57). Once the virus is transferred to the tissues, it initiates the pre-acute phase of infection, characterized by an inflammatory response, including increased permeability and chemokine release from resident cells. After viral replication (**Figure 1**), it systemically migrates through lymph nodes, joints, spleen, liver, brain and muscles (58–61). During the initial days of infection, high titres of the virus can be detected in the blood (62–65).

The innate immunity plays an important role in controlling arboviruses in the early stages of infection. As is well known, the viral RNA is a pathogen-associated molecular patterns (PAMP) recognized by pattern recognition receptors (PRRs), mainly Toll-like receptors (TLRs 3, 7 and 8), RIG-I like receptors (RLRs) and Nod-like receptors (NLRs) (66, 67). As with other viral infections, type I interferon (IFN-α and IFN-β) acts as the first line of defense against arboviruses. These interferons stimulate the expression of many genes that interupt the virus replication and proliferation (68, 69). The IFNs activate the cells through a signal transducer such as Janus kinase, which induces the transcription factor JAK-STAT to produce interferon regulatory factors (IRFs 3, 5 and 7) and interferon-stimulated genes (ISG), that block viral replication by triggering an antiviral state in both infected and uninfected cells (70–72). Secondary infections, however, are known to cause serious diseases, specifically after a heterotypic infection (73). It is believed that the phenomenon of antibody-dependent enhancement (ADE) may cause increased virulence and pathogenicity (74).

The ADE phenomenon occurs when non-neutralizing antibodies from a previous heterotypic infection create a virus-antibody complex that is phagocytosed by cells that are generally not infected, via Fcγ receptors and complement receptors present in permissive cell for ADE, such as dendritic cells, monocytes and macrophages (75). ADE is primarily mediated by the IgG antibody, and in some instances, by IgM (76). It is expected to result in an increase in viral uptake and, therefore, contribute to the replication, leading to an exacerbated immune response to the infection, and cytokine storm, with an upregulation of IL-6, TNF-α and IL-10, whereas IL-12 and IFN-γ is downregulated (77).

It is worth mentioning Vo et al. (78) that conducted a study to investigate the phenomenon of ADE in the context of the four DENV serotypes by assessing plasma samples from patients with confirmed DENV-2 infections. Initially, during the early stages of infection, no significant differences in ADE activity were observed among the serotypes. However, intriguing distinctions emerged in later stages, specifically at 10 and 60 days post-infection confirmation. ADE activity was found to be notably higher against DENV-1 when compared to DENV-4 during these later time points. When analyzing the cumulative ADE activity by calculating the area under the curve (AUC), it was determined to be most pronounced against DENV-2, which happened to be the serotype responsible for the infection in the cohort under investigation (78).

Moreover, overall it is not new that DENV antibody avidity shifts from the previous infecting serotype to the current infecting serotype over time (79). Over time, there is a transition in the overall avidity of these antibodies, influenced by changing quantities and qualities. For example, the quantity of afucosylated Fc-IgG increases during convalescence compared to the acute phase of primary infections, potentially impacting the occurrence of ADE (80, 81). Additionally, antibody titers specific to DENV tend to decrease as time progresses, which may further contribute to the susceptibility to ADE (82, 83). This is of particular concern since antibodies targeting specific regions of viral proteins, such as the fusion loop of the envelope protein (E) and other surface proteins like (prM), have been identified as factors promoting ADE both in laboratory settings and animal models (84, 85). Structural aspects of the virus, including its maturation state, have also been found to influence ADE. Notably, severe cases of DENV infection can witness an increase in mortality rates of up to 20% (73).

Another important factor for pathogenicity is the NS1. This protein is found on the surface of infected cells and circulating viral particles in the host. The interaction of NS1 with cellular receptors and viral proteins contributes to viral replication, viable viral particles, viral persistence, and primarily the activation of immune system receptors, such as C3, C4, and C5 complement receptors, and even the membrane attack complex (86). TLR3, present in dendritic cells and macrophages, is activated via NS1, triggering the expression of antiviral factors. NS1 also activates TLR2 and TLR6 during DENV infection, increasing the production of proinflammatory cytokines like IL-6 and TNF-α (87). The recognition of NS1 by TLR4 causes the activation of peripheral blood mononuclear cells (PBMCs), leading to an increase in proinflammatory cytokines that induce endothelial tissue dysfunction (88). Studies using human endothelial cell cultures have shown that flavivirus NS1 can cause modulation and endothelial hyperpermeability in various tissues, favoring pathogenesis (89).

The most severe dengue clinical syndrome can manifest itself in the form of shock, including coagulation abnormalities, plasma leakage, and increased vascular fragility. Fluid loss due to increased capillary permeability leads to hypovolemic shock and multiorgan failure (90). Because the clinical diagnosis of these diseases is not specific (91), due to the wide spectrum of clinical manifestations and clinical overlap with other circulating arboviruses, molecular diagnostic techniques are necessary and are the most commonly used to confirm infection by some flavivirus at the beginning of symptoms (92, 93).

The presence of viral RNA in the amniotic fluid and placenta evidences the association of Congenital Zika Syndrome (SCZ) with neonatal complications, such as congenital microcephaly, optic neuropathy, congenital glaucoma, ventriculomegaly and lissencephaly (94, 95). Beyond neonatal complications, ZIKV infection in adults is related to Guillain Barré Syndrome (GBS), an autoimmune disease that attacks neural cells leading to gradual muscle weakness and even paralysis. Other complications such as arthralgia and cardiovascular problems have been reported; however, it is necessary to establish the exact connection (96, 97).

In the chronic phase, CHIKV propagation through the lymphatic, circulatory, and joint systems (98). The chronic-phase arthritis and the pro-inflammatory environment are associated, the persistence of viral RNA in macrophages, muscles and joints may favor persistent arthritis (99). Unlike other arboviruses, which show symptoms only in the acute infection phase, more than a third of CHIKV-caused infections are symptomatic and most patients experience joint pain years after the onset of the disease (100, 101).

## 4 Outlook on control strategies development

It is necessary to understand in detail the Arbovirus’ biology, its relationship with the vectors, with the host and its history, so that is possible to develop integrated control strategies in an effective way, because multiple factors contribute to the emergence of arboviruses. For instance, population growth in areas with unplanned urbanization, climate changes, and viral genetic adaptation are significant contributors (30). Beyond their transmission between arthropods and humans, these viruses can infect a wide range of animal species, rendering complete eradication virtually impossible. Arbovirus infections exhibit diverse clinical presentations, ranging from mild to severe, and are categorized as either visceral or neurotropic, with some viruses displaying both characteristics (102, 103).

### 4.1 Vaccines

An effective vaccine against DENV must be able to induce a balanced response against all four DENV serotypes (104). However, its development is challenging due to the theoretical risk that an incomplete immunity generated by the vaccine could lead to increased pathogenesis upon subsequent natural infection, as discussed earlier with the ADE phenomenon.

Dengvaxia^®^ (Sanofi Pasteur) is a live attenuated tetravalent vaccine containing chimeras of pre-structural membrane (prM) and envelope (E) genes from the four DENV types, combined with non-structural genes from the yellow fever vaccine strain 17D (dengue chimeric yellow fever – CYD) (105). It has been licensed in several American countries, including Mexico, Brazil, El Salvador, Costa Rica, Paraguay, Guatemala, and Peru (106). However, despite being licensed by ANVISA (The National Health Surveillance Agency) in 2015 (107), Dengvaxia^®^ has raised numerous concerns in the scientific and health communities, particularly regarding its potential to predispose people with no prior DENV exposure to severe diseases. As a result, the vaccine is no longer considered a viable prevention tool, partly due to its high cost (106).

In the Philippines, immunizations with Dengvaxia^®^ were suspended in 2017 after 14 vaccination-associated child deaths occurred (108). In Brazil, the vaccine was recommended for individuals aged 9 to 45 years old, with a documented history of prior laboratory-confirmed DENV infection (106). In the European Union and the United States, Dengvaxia^®^ was also licensed for its use in people aged 9 or older living in DENV endemic areas, but limited to those who had experienced a previous DENV infection (109, 110). The Advisory Committee on Immunization Practices (ACIP) recommended in June 2021 Dengvaxia for routine use in children aged 9 to 16 years living in endemic areas with laboratory confirmation of previous DENV infection (111). Low acceptance of the vaccine is driven by concerns about an increased risk of severe dengue in vaccinated individuals without prior exposure to DENV.

Recently, the ANVISA approved the QDENGA^®^ vaccine (TAK-003 – Takeda Pharma) to immunize individuals aged 4 - 60 years, without the need to confirm a previous infection, different from the Dengvaxia^®^ vaccine. This vaccine, QDENGA^®^, is based on a live attenuated DENV-2 virus that serves as the genetic backbone for the four vaccine viruses, the chimeras (DENV-1, DENV-3, and DENV-4), which were generated by replacing the pre-membrane and envelope genes. The QDENGA^®^ vaccine also received evaluation and a positive recommendation from the European Health Agency (EMA) under the “EU Medicines for all” program. Several clinical trials involving more than 20,000 subjects have demonstrated the vaccine’s safety and efficacy against DENV disease (112–114).

Despite the success of vaccines already licensed for certain flaviviruses such as Yellow Fever Virus (YFV), and Japanese Encephalitis Virus (JEV), combating epidemics caused by emerging flaviviruses poses significant challenges. The primary challenge stems from the extensive cross-reactivity observed in flavivirus-immune sera. While neutralization assays help to understanding antibody responses to both homologous and heterologous viruses in convalescent sera, the use of sera from acute infected individuals has been limited (115, 116).

The detection of flavivirus infection using molecular assays is limited due to the transient nature of viremia, making them sensitive only for relatively short periods. To develop vaccines against flaviviruses, new platforms must be explored, underscoring the necessity for further studies on the biology, structure, and heterogeneity of vaccine antigens (117).

The accessibility to vaccines also remains a significant challenge even after their development. This issue is particularly evident in regions like South America and Africa, where vaccine shortages have led to a continuous circulation of Yellow Fever Virus (YFV), prompting research on vaccine economics (118). Furthermore, the availability of effective vaccines does not always guarantee the expected impact on global health. An illustrative example was observed during the SARS-CoV-2 pandemic, where in many areas, the acceptance of vaccination was lower than anticipated, necessitating the implementation of public policies to ensure access and raise awareness about the importance of this preventive measure.

High safety, immunogenicity, and efficacy are always essential prerequisites for a vaccine to be embraced by both the public and the scientific community. However, a crucial question remains unanswered: Will these DENV vaccines offer superior protection to individuals with no prior exposure to DENV infections? Ensuring a vaccine’s effectiveness without the risk of sensitizing the population and exposing them to symptomatic or severe disease upon subsequent natural DENV infection is of utmost importance. Therefore, addressing these questions is imperative before we can consider using these vaccines as a significant tool in the prevention and control of dengue disease on a public scale.

**Figure 4:**
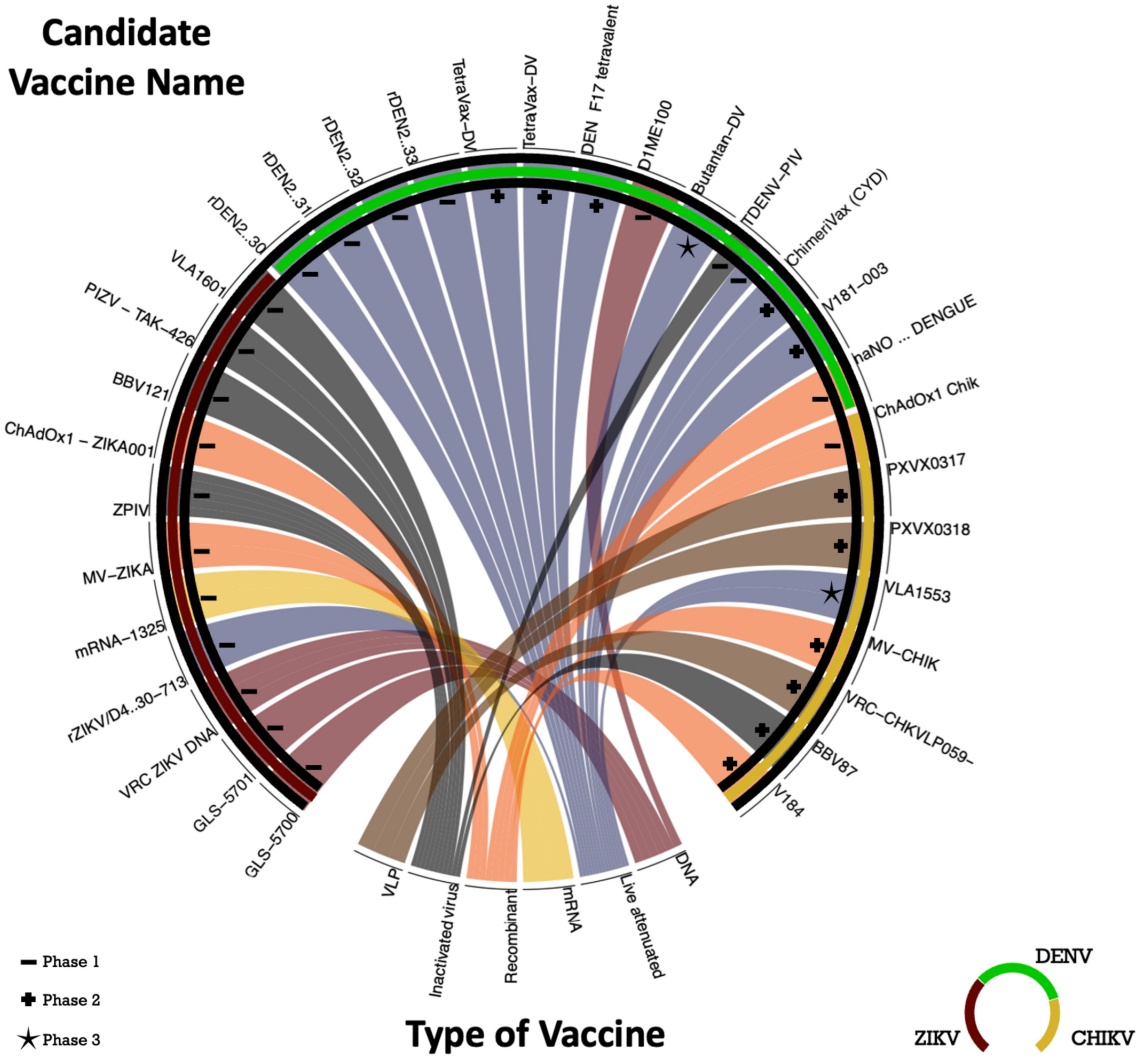
Chord Diagram with candidate vaccine in clinical trials to DENV, ZIKV and CHIKV. The chord diagram employed in this study depicts a central circle representing the target virus of the vaccines, including DENV (dark green), ZIKV (red), and CHIKV (yellow). The diverse segments of the circle are assigned to distinct vaccine development methodologies: 3 trials on VLP (brown), 5 trials on inactivated virus (gray), 6 trials on recombinant methods (orange), 1 mRNA trial (yellow), 12 trials on live attenuated (blue), and 1 trials involving DNA (red). The phases of the clinical trials are indicated by symbols: dash (Phase 1), plus (Phase 2), and star (Phase 3). The vaccines mentioned in the chord diagram are registered in the ClinicalTrials.gov - National Library of Medicine (U.S.) database.

Currently, no vaccines are available to prevent ZIKV and CHIKV infections. Nevertheless, several vaccine candidates are in the developmental pipeline (see **Figure 4**), with ongoing clinical trials. These vaccines utilize diverse technologies, encompassing live attenuated, inactivated virus, mRNA, DNA, recombinant, and VLP approaches.

### 4.2 Antivirals therapies

At the present, there are no specific therapy against arbovirus-causing infections, but some drugs are used to reduce symptoms (**Table 1**). Studies using “sofosbuvir” as an antiviral agent has been shown to inhibit ZIKV replication in hepatic and neural cell cultures, and also provided protection using animal model such as mice after being challenged with ZIKV (119). In another study, the antibiotic azithromycin was analyzed *in vitro* using glial cells, and it was capable to reduce viral proliferation (120). Even though these data are promising, it is known that the ideal drug against ZIKV should reduce viral load, symptoms, and prevent neurological complications in the fetus, what these experiments are far from achieving it (121, 122).

**Table 1:** Possible antiviral targets and mosquito control strategies.

IMPLEMENTATION OF PREVENTIVE MEASURES	
Antiviral Proactiveness – Pre infection	
Strategy	Description
**Metatranscriptomics surveillance**	Monitors arboviruses in mosquitoes, mutations identification and transmission
**Genetically modified mosquitoes**	The mosquito’s natural defense mechanisms allow new strategies for insect resistance to viruses
**Mosquito bacterial microbiota**	Promotes a reduction in the viral replication capacity of vectors.
Prophylaxis - post infection	
**Name/Compound **	Mode of action
**Sofosbuvir**	Inhibits viral replication **(ZIKV)**
**Azithromycin**	Inhibits viral replication **(ZIKV)**
**Ribavirin**	Antiviral activity **(CHIKV)**
**Chloroquine**	Antiviral activity **(CHIKV)**
**Niclosamide**	Antiviral activity **(DENV, ZIKV and CHIKV)**
**Spiropyrazolopyridone**	Antiviral activity **(DENV)**
**1-[(2-methylbenzimidazol-1-yl) methyl]-2-oxo-indolin-3-ylidene]-amino] thiourea**	Antiviral activity **(CHIKV)**
**Delphinidin**	Antiviral activity in early stages **(DENV and ZIKV)**
**Epigallocatechin gallate**	Inhibits infection and antiviral activity **(DENV and CHIKV)**

In the case of CHIKV, the treatment of arthralgia often involves the use of non-steroidal anti-inflammatory drugs, such as ribavirin and chloroquine. Additionally, the investigation of monoclonal antibodies as potential therapeutic agents has been explored (123–125). Passive transfer of immune serum protects against virus-induced lethality and studies in mice demonstrated a prophylactic efficacy when it is provided before or immediately after CHIKV challenge (124). Ribavirin has antiviral activity against several RNA viruses, and the authors (126, 127) suggest that it has a beneficial effect in relieving arthralgia and swelling associated with chronic arthralgia. Chloroquine is another treatment that has been shown to be effective as it inhibited CHIKV infection in cultured cells through endosomal acidification, thus interfering with the activation of Toll-like receptors (TLR), reducing inflammatory activity (128–130). Niclosamide, a medication used as an antiparasitic, was able to inhibit *in vitro* the entry of DENV, ZIKV, and CHIKV into the cells. This medication also interferes with endosomal acidification and inhibits membrane fusion (131, 132).

Isatin and its derivatives are heterocyclic organic compounds. They have broad applications in the pharmacological field, and several studies have pointed out their antiviral activities, such as spiropyrazolopyridone. Bin Zou and colleagues (133) used a viral infection model in mice with DENV and observed a reduction in viremia in the treated mice after oral administration of the drug. Another isatin derivative, 1-[(2-methylbenzimidazol-1-yl)methyl]-2-oxo-indolin-3-ylidene]-amino]thiourea (MBZM-N-IBT), demonstrated antiviral activity against CHIKV by inhibiting the nsP2 protease activity *in vitro* and *in vivo* experiments (134, 135).

Natural compounds belonging to the Flavonoid family, such as Delphinidin (D) and epigallocatechin gallate (EGCG), possess antioxidant activities, and according to recent studies, they also exhibit antiviral functions against some arboviruses, such as DENV, ZIKV, and CHIKV. Delphinidin likely acts during the interaction of viral proteins with receptors present in cells; this compound showed antiviral activity only during the early stages of viral infection tested *in vitro* (136). The EGCG was also able to inhibit CHIKV infection and attchament to cells, as shown by researchers (137) that transfected HEK cells with lentiviral vectors pseudotyped with CHIKV envelope proteins and subsequently added EGCG to assess antiviral effects.

### 4.3 Vector control strategies

Although there are several important studies in progress, the absence or limitation of effective therapies and vaccines makes the control of circulating mosquitoes vectors a commonly used strategy to combat arbovirus transmission. Vector control represents a viable approach to reduce the prevalence of these disease vectors (**Table 1**). Arboviruses are distributed worldwide, and their surveillance has become a crucial tool for detecting and controlling the circulation of viruses (138). Metatranscriptomics surveillance programs monitor and test mosquito populations for arboviruses, as demostrated by The Victorian Arbovirus Disease Control Program (VADCP) (139). Metatranscriptomics is a novel RNA sequencing approach used to analyze the RNA present in a sample. This technique aids in identifying viral mutations and even tracing the origin of cases. To achieve it, a complex bioinformatic analysis is required, as demonstrated in a study conducted in Australia (140). In this study, they analyzed the species of mosquitoes and the circulating virus to gain valuable insights into viral dynamics and transmission patterns.

Various methods targeting different stages of the mosquito life cycle can aid in controlling their population. Implementation of these strategies involves public education and raising awareness, encouraging measures like managing and preventing the deposition of eggs and larvae in water accumulations, tires, plant pots, and uncovered water tanks. Additionally, the application of insecticides to eliminate mosquito larvae and adults is crucial (141).

Furthermore, the incorporation of antiviral controls within mosquitoes has shown promise in preventing arbovirus transmission. Some recent studies have explored the use of genetically modified mosquitoes with antiviral genes that hinder viral replication (142).

Mosquitoes present a different defense system compared to mammals; they lack B and T lymphocytes, do not produce immunoglobulins, and do not develop specific antigen responses. However, they do exhibit defense mechanisms found in the innate immunity of mammals, such as Toll-like pathways, Janus kinase/signal transducer and activator of transcription (JAK/STAT), immune deficiency (IMD), and RNA interference (RNAi) pathways, which interfere with viral gene expression (143).

The Toll-like receptors (TLRs) bind to cytokine receptors like Spätzle, inducing signal transductions in the cytoplasm, where they interact with the myeloid differentiation primary response protein (MyD88)-Tube-Pelle complex, leading to the expression of B cell-dependent immune response-related NF-kB. The NF-kB expression can also be stimulated via IMD through the activation of peptidoglycan-recognition protein receptors PGPR-LC and PGPR-LG. Activation of the JAK/STAT pathway initiates the transcription of antiviral cytokines and growth factors.

Gene regulation is carried out by small non-coding RNAs, approximately 21-30 nucleotides long, classified as small interfering RNAs (siRNAs), microRNAs (miRNAs), and Piwi-associated interfering RNAs (piRNAs) (144, 145). siRNAs are double-stranded RNA molecules that target complementary messenger RNA (mRNA) sequences, subsequently degrading the gene and suppressing its function. miRNAs act in the post-transcriptional phase, repressing mRNA translation, while piRNAs regulate element transposition (144–147).

A better understanding of these insect modulation mechanisms aids in developing more efficient approaches for arbovirus control. This includes inhibiting specific genes to reduce virus transmission, enhancing insect resistance to the virus by using antimicrobial peptides, and even inhibiting viral replication in mosquitoes to prevent transmission.

Currently, a cutting-edge methodology for arbovirus control involves the manipulation of mosquito bacterial symbionts’ microbiota. The composition of the bacterial community can vary among different mosquito species (141). One notable example is *Wolbachia pipiens*, an α-proteobacterium found in a few arthropod species, which has been extensively researched for insect population control. *Wolbachia* is present in various mosquito tissues, including the Malpighian tubules, muscles, head, glands, and reproductive organs (148). The technique employed to introduce *Wolbachia* into insects is termed Insect Lineage Infection by *Wolbachia*. This method entails inoculating *Wolbachia* bacteria into insect eggs during the embryonic stage, resulting in the establishment of a persistent infection in the cells.Its effective spread is attributed to its ability to induce cytoplasmic incompatibility (CI), where mating between uninfected females and *Wolbachia*-infected males results in eggs that fail to develop, leading to the production of only *Wolbachia*-infected offspring. Additionally, *Wolbachia* can cause reduction of vectorial capacity by decreasing the mosquito’s lifespan (parthenogenesis) and inducing feminization. Moreover, *Wolbachia* inhibits virus replication within the mosquito and decreases saliva production, affecting feeding capacity during meals (149–151).

Research conducted in Brazil and Colombia has shown promising results in vector control, demonstrating that the release of modified *Aedes aegypti* mosquitoes containing *Wolbachia* led to the suppression of arbovirus transmission and replication. Consequently, *Wolbachia* has been proven capable of inhibiting the replication of DENV, ZIKV, and CHIKV (152–154). The use of *Wolbachia* as a vector control agent has been applied in several countries as part of programs aimed at eliminating arboviruses. One such example is the “Eliminate Dengue” project, an international program with the objective of controlling DENV circulation through *Wolbachia* infection in *Aedes aegypti* mosquitoes. This program has been implemented in countries such as Australia, Brazil, Indonesia, Vietnam, and Colombia, showing promising results in reducing DENV transmission rates. The initiative that combats the spread of arboviruses using *Wolbachia* is the “World Mosquito Program” (WMP). These prevention and control programs, which are based on the introduction of *Wolbachia* into insects, represent a promising and innovative approach (155).

## 5 Conclusion

The reemergence of arbovirus infections has been observed in several countries. Furthermore, the spread to new geographical areas is on the rise, underscoring the necessity for the implementation of effective control and prevention programs. These programs should be grounded in entomological surveillance activities and disease monitoring.

While specific pharmaceutical interventions for arbovirus infections remain elusive, we conclude that encouraging progress has been made with certain compounds, including Chloroquine, Niclosamide, and Isatin derivatives. These compounds have demonstrated substantial antiviral efficacy against these pathogens in both laboratory and animal studies.

It is crucial to implement strategic vector control measures to contain arbovirus transmission. These strategies encompass a range of interventions, as exemplified by the VADCP, along with educational campaigns to raise public awareness. Notably, advanced techniques like the manipulation of bacterial symbionts in mosquitoes, illustrated by the utilization of *Wolbachia* in *Aedes aegypti* mosquitoes, have shown remarkable success in suppressing the transmission of diseases like DENV across various nations, which holds potential such as pivotal component of integrated prevention strategy. By reducing vector competence, limiting viral replication, and influencing mosquito lifespan, *Wolbachia* contributes substantially to breaking the transmission cycle.

As we confront the complex challenges posed by arboviruses, it becomes evident that a holistic and collaborative approach is imperative. The integration of advancements in antiviral research, the development of effective vaccines, innovative vector control methodologies, and surveillance programs collectively fortify the global defense. This unified front not only prevents immediate outbreaks, but also lays the foundation for resilient and adaptative aproch to combat future arbovirus challegens.

## Author contributions

NC: Conceptualization, Writing – original draft. AL: Conceptualization, Writing – original draft. WP: Writing – review & editing. JS: Writing – review & editing. LV: Writing – review & editing. WC: Writing – review & editing. AF: Writing – review & editing. OC: Writing – review & editing. GC: Writing – review & editing. RD: Writing – review & editing.
